# Discovery of natural apigenin analogues as lysine-specific demethylase 1 inhibitors against tumoral testicular germ cells

**DOI:** 10.1038/s41598-026-42263-y

**Published:** 2026-03-02

**Authors:** Li-Wei Sun, Meng Zhang, Cai-Fang Li, Cong Wang, Yang Li

**Affiliations:** Xinxiang Central Hospital, The Fourth Clinical College of Henan Medical University, Xinxiang, 453000 China

**Keywords:** Natural apigenin analogues, Testicular germ cell tumors, Lysine-specific demethylase 1, Structure activity relationships, Proliferation, Biochemistry, Cancer, Drug discovery

## Abstract

Testicular germ cell tumors (TGCT) pose a threat to men’s health, discovery of small molecule compounds with potent antiproliferative activity against testicular germ cells is of great significance. Lysine-specific demethylase 1 (LSD1) is overexpressed in tumoral testicular germ cell lines, and it is a promising target for developing agents against TGCT. A series of natural apigenin analogues were evaluated for their inhibitory activity against LSD1, and their structure activity relationships (SARs) were explored. Among them, 8,3’-diprenylapigenin exhibited the most potent inhibitory activity against LSD1 with an IC_50_ value of 3.60 µM. 7-hydroxy of 8,3’-diprenylapigenin formed a hydrogen bond with Ala809 of LSD1, demonstrating that 7-hydroxy was a dominant group. In addition, 8,3’-diprenylapigenin reversibly and selectively inhibited LSD1 in a concentration-dependent and time-dependent manner. It inhibited proliferation against tumoral testicular germ cell lines NCCIT and NTERA-2 with IC_50_ values of 9.37 µM and 5.26 µM, respectively. Mechanism studies revealed that 8,3’-diprenylapigenin induced the generation of ROS, inhibited the activity of catalase and decreased the level of ATP in NTERA-2 cells. Meanwhile, it also activated the release of LDH, increased the activity of SOD and enhanced the level of MDA in NTERA-2 cells. These findings indicated that 8,3’-diprenylapigenin is a novel reversible LSD1 inhibitor and deserves further exploration to treat testicular germ cell tumors.

**Supplementary Information:**

The online version contains supplementary material available at 10.1038/s41598-026-42263-y.

## Introduction

Testicular germ cell tumors (TGCT) are the most common malignant tumors of the male testicles, which are classified into seminomas and non-seminomas^1–3^. Over 50% of TGCT patients suffer from oligospermia, while 6%−24% of TGCT patients have azoospermia^4^. In addition, radiotherapy, surgery and adjuvant chemotherapy result in 60% of TGCT patients suffering from azoospermia^5–7^. Because many TGCT patients hope to have children normally, the preservation of fertility is an important consideration^8^. Therefore, discovery of novel therapeutic agents with potent antiproliferative effects against tumoral testicular germ cells and weak cytotoxic effects against normal testicular cells is very necessary^9–11^.

**Fig. 1:**
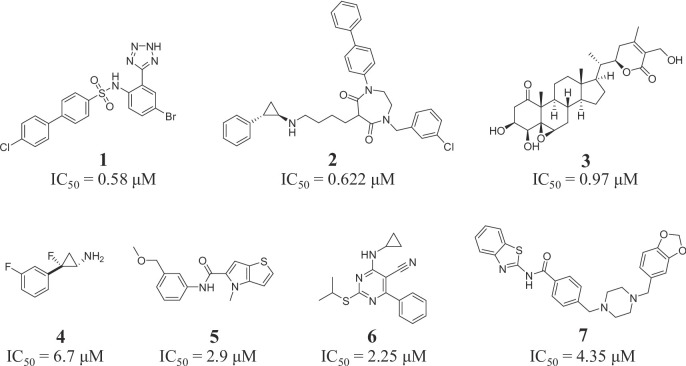
Chemical structures and enzymatic activity of LSD1 inhibitors.

Lysine-specific demethylase 1 (LSD1), as a demethylase, is highly expressed in various cancers such as prostate cancer, gastric cancer, and esophageal cancer^12–15^. In recent years, many LSD1 inhibitors with different structural types have been developed as anti-tumor agents^16–19^. Tetrazole derivative **1** (Fig. 1) as a reversible LSD1 inhibitor with an IC_50_ value of 0.58 µM could upregulate the expression level of H3K4me2 in neuroblastoma cells^20^. 1,4-Diazepane-5,7-dione derivative **2** was a selective LSD1 inhibitor with an IC_50_ value of 0.622 µM, and it exhibited the antiproliferative activity against A549 cells with an IC_50_ value of 5.8 µM^21^. Steroid analogue **3** was a selective LSD1 inhibitor with an IC_50_ value of 0.97 µM, and it could inhibit proliferation against C42B cells with an IC_50_ value of 3.86 µM^22^. Fluorinated tranylcypromine **4** showed potent inhibitory activity against LSD1 with an IC_50_ value of 6.7 µM^23^. 4*H*-thieno[3, 2-*b*]pyrrole analogue **5** was a LSD1 inhibitor with an IC_50_ value of 2.9 µM^24^. Pyrimidine derivative **6** was a potent LSD1 inhibitor with an IC_50_ value of 2.25 µM, and suppressed proliferation against HCT-116 cells with an IC_50_ value of 8.78 µM^25^. Benzothiazole analogue **7** was a potent LSD1 inhibitor with an IC_50_ value of 4.35 µM^26^. Importantly, recent studies reported that LSD1 was overexpressed in tumoral testicular germ cell lines NCCIT and NTERA-2^27^. Therefore, LSD1 may be a novel target to develop small molecule inhibitors against testicular germ cell tumors.

Apigenin (4’,5,7-trihydroxyflavone) was a natural flavonoid, which could be isolated from various fruits and vegetables^28–31^. Although apigenin derivatives have been reported to exert diverse bioactivities^32–34^, their antiproliferative effects against testicular germ cell tumors remain unclear. The discovery strategy of apigenin analogues as LSD1 inhibitors was shown in **Fig. ****S1**. The preliminary enzymatic activity screening against LSD1 of all natural products in our lab were performed, and we found that apigenin displayed the inhibitory activity against LSD1 with an IC_50_ value of < 20 µM. Therefore, we collected apigenin analogues and established a natural apigenin library in this work. Based on the enzymatic activity screening for natural apigenin analogues, 8,3’-diprenylapigenin was first identified as a novel LSD1 inhibitor with an IC_50_ value of 3.60 µM. Meanwhile, 8,3’-diprenylapigenin displayed potent antiproliferative activity against tumoral testicular germ cells and exhibited weak cytotoxic effects against normal testicular cells. Importantly, structure activity relationships, molecular docking and potential mechanisms of 8,3’-diprenylapigenin were investigated.

## Methodology

### The source of natural apigenin analogues

In this work, apigenin, 3,6-dimethoxyapigenin, apigenin-7-glucuronide, apigenin 7-O-methylglucuronide, trimethylapigenin, 8,3’-diprenylapigenin, 7,4’-di-O-methylapigenin, apigenin 7-diglucuronide, 8-hydroxyapigenin, apigenin 7,4’-diglucoside, 6,8-dimethylapigenin, apigenin 7-O-glucoside, apigenin 7-O-rutinoside and apigenin 7-neohesperidoside were purchased from ChemFaces (Wuhan, China), and their product number were CFN98843, CFN97961, CFN98500, CFN98894, CFN91890, CFN96216, CFN98819, CFN91599, CFN91491, CFN95648, CFN96061, CFN98981, CFN98927 and CFN99814, respectively. Apigenin triacetate and 3’,3’’’-biapigenin were purchased from MedChemExpress (Shanghai, China), and their product number were HY-W748594 and HY-N9806, respectively.

### Enzymatic activity against LSD1

Horseradish peroxidase (6 U/mL), Amplex Red (20 nM), FAD (50 nM), H3K4me2 peptide (25 µM), recombinant LSD1 (5 nM) and natural apigenin analogues at different concentrations were incubated for 0.5 h. The fluorescence values of natural apigenin analogues were detected at em 590 nm and ex 530 nm using EnSpire (PerkinElmer, Massachusetts, America). In this assay, LSD1-IN-24 (MedChemExpress, Shanghai, China) as a LSD1 inhibitor was used as a control agent. All enzymatic assays were performed in triplicate, and three independent experiments were conducted. Data are expressed as mean ± standard deviation (SD).

### Dialysis assay

20 µM of 8,3’-diprenylapigenin was incubated with the recombinant LSD1 at 37 °C for 1.5 h. The system was dialyzed at 4 °C for 24 h in the presence of HEPES buffer (50 mM, pH 7.96). Then, HEPES buffer was replaced twice a day. Finally, the inhibitory activity of LSD1 was detected. In the dialysis assay, GSK-LSD1 (MedChemExpress, Shanghai, China) as an irreversible LSD1 inhibitor was the control agent. The dialysis assay was performed in three independent experiments, and data are expressed as mean ± SD.

### Enzymatic activity against MAO-A/B

MAO enzymes were purchased from Shanghai Aiyue Biotechnology Co., LTD (Shanghai, China), and the enzymatic selectivity of 8,3’-diprenylapigenin was explored. Luciferin substrate (10 µM), MAO-A or MAO-B (20 nM) and 8,3’-diprenylapigenin at different concentrations (2.5 µM, 5 µM and 10 µM) were incubated at 37 °C for 70 min. Then, luciferase (20 µL) was added and the system was incubated at 37 °C for 20 min. Luminescence was detected by a plate reader purchased from Thermo Scientific (Massachusetts, America). All MAO-A/B enzymatic assays were performed in triplicate with three independent experiments. Data are expressed as mean ± SD.

### Cell viability assay

NCCIT, NTERA-2, TM3 and TM4 cell lines were purchased from Wuhan Ponose Life Science and Technology Co., LTD (Wuhan, China). Cells were cultured in 10% fetal bovine serum (Thermo Scientific, Massachusetts, America) at 37 °C. In the cell viability assay, cells were cultured in a 96-well plate and 8,3’-diprenylapigenin was treated for 48 h. 10 µL of CCK8 (Servicebio, Wuhan, China) was added into each well and incubated at 37 °C for 2 h. The code of CCK8 kit was G4103-5ML. The absorbance value was detected at 490 nm by a plate reader. Cell viability assays were performed in triplicate, and three independent experiments were conducted. IC₅₀ values were calculated using GraphPad Prism software, and data are expressed as mean ± SD.

### Detection of ROS

ROS detection kit was purchased from Servicebio (Wuhan, China), and the code of ROS detection kit was G1706-100T. NTERA-2 cells were cultured into a 6-well plate at 37 °C. Different concentrations of 8,3’-diprenylapigenin were added and incubated at 37 °C for 48 h. After the system was washed by PBS, DCFH-DA solution was added and incubated in the dark at 37 °C for 30 min. NTERA-2 cells were observed by a fluorescence microscope (Thermo Scientific, Massachusetts, America).

### Detection of catalase activity

Catalase detection kit was purchased from Servicebio (Wuhan, China), and the code of catalase detection kit was G4307-48T. NTERA-2 cells were treated with 8,3’-diprenylapigenin for 48 h at 37 °C. The supernatant was obtained by centrifuging NTERA-2 cells and was incubated in the chromogenic working solution for 10 min. The absorbance value was detected at 405 nm by a plate reader. Catalase activity assays were performed in triplicate with three independent experiments. Data are expressed as mean ± SD.

### ATP content assay

ATP content detection kit was purchased from Servicebio (Wuhan, China), and the code of ATP content detection kit was G4309-48T. NTERA-2 cells were cultured into a 6-well plate at 37 °C. Different concentrations of 8,3’-diprenylapigenin were added and incubated at 37 °C for 48 h. The supernatant was obtained by centrifuging NTERA-2 cells and was incubated in the ATP working solution for 15 s. Luminescence was detected by a plate reader purchased from Thermo Scientific (Massachusetts, America). ATP level measurements were performed in triplicate with three independent experiments. Data are expressed as mean ± SD.

### LDH leakage assay

NTERA-2 cells were cultured in a 96-well plate, and 8,3’-diprenylapigenin at different concentrations were added for 24–48 h. Then, cells in the each well was centrifuged at 1000 g for 5 min and the supernatant was obtained to measure the lactate dehydrogenase. Next, LDH working solution (Servicebio, Wuhan, China) was added and incubated in the dark at 37 °C for 30 min. The absorbance value was detected at 490 nm by a plate reader. LDH activity assays were performed in triplicate with three independent experiments. Data are expressed as mean ± SD.

### Detection of SOD activity

SOD activity detection kit was purchased from beyotime (Shanghai, China), and the code of SOD activity detection kit was G4306-48T. NTERA-2 cells were treated with 8,3’-diprenylapigenin for 24–48 h. The supernatant was obtained by centrifuging NTERA-2 cells. 151 µL of SOD detection buffer, 8 µL of WST-8 solution, 1 µL of SOD enzymatic solution and 20 µL of 8,3’-diprenylapigenin solution were incubated at 37 °C for 30 min. The absorbance value was detected at 450 nm by a plate reader. SOD activity assays were performed in triplicate with three independent experiments. Data are expressed as mean ± SD.

### MDA assay

Malondialdehyde detection kit was purchased from beyotime (Shanghai, China), and the code of malondialdehyde detection kit was G4300-48T. NTERA-2 cells were treated with 8,3’-diprenylapigenin for 24–48 h. The supernatant was obtained by centrifuging NTERA-2 cells to measure the MDA level. MDA detection working solution and the supernatant were incubated at 100 °C for 15 min. The system was centrifuged at 1000 g for 10 min to obtain the new supernatant. The absorbance value was detected at 530 nm by a plate reader. MDA level measurements were performed in triplicate with three independent experiments. Data are expressed as mean ± SD.

### Statistical analysis

All experiments were repeated three times, and data were expressed as the mean ± standard deviation of these three independent experiments. Graphpad prism 10 (GraphPad Software, LLC, San Diego, America) was used to calculate the IC_50_ values. ^*^P < 0.05, ^**^P < 0.01, ^***^P < 0.001 and ^****^P < 0.0001 were considered a statistically significant difference.

## Results

### Natural apigenin analogues were potential LSD1 inhibitors

A series of natural apigenin analogues were evaluated for their inhibitory effects against LSD1. Their enzymatic activity and chemical structures of natural apigenin analogues were shown in Table 1. LSD1-IN-24 as a LSD1 inhibitor was used as a control agent. In this work, apigenin, 3,6-dimethoxyapigenin, apigenin-7-glucuronide, apigenin 7-O-methylglucuronide, trimethylapigenin, 8,3’-diprenylapigenin, 7,4’-di-O-methylapigenin, apigenin 7-diglucuronide, apigenin triacetate, 3’,3’’’-biapigenin, 8-hydroxyapigenin, apigenin 7,4’-diglucoside, 6,8-dimethylapigenin, apigenin 7-O-glucoside, apigenin 7-O-rutinoside and apigenin 7-neohesperidoside displayed the inhibitory activity against LSD1 with IC_50_ values of 13.42 µM, 8.75 µM, > 40 µM, > 40 µM, 27.63 µM, 3.60 µM, 15.93 µM, 30.51 µM, 38.67 µM, 19.27 µM, 10.09 µM, > 40 µM, 19.76 µM, 21.39 µM, 32.67 µM and > 40 µM, respectively. These findings indicated that natural apigenin analogues were potential LSD1 inhibitors.

**Table 1:** Enzymatic activity and chemical structures of natural apigenin analogues.

No.	Compound name	Chemical structure	IC_50_ (µM)
1	apigenin	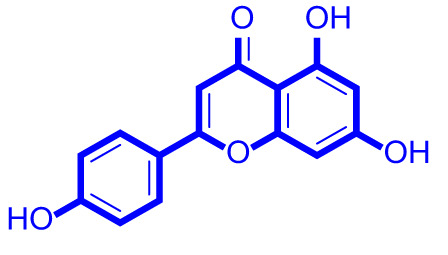	13.42 ± 0.63
2	3,6-dimethoxyapigenin	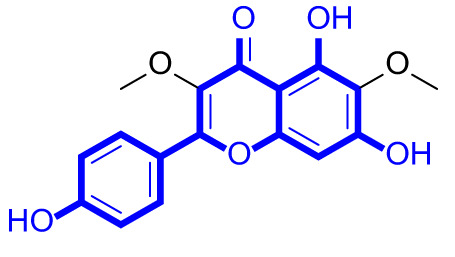	8.75 ± 0.98
3	apigenin-7-glucuronide	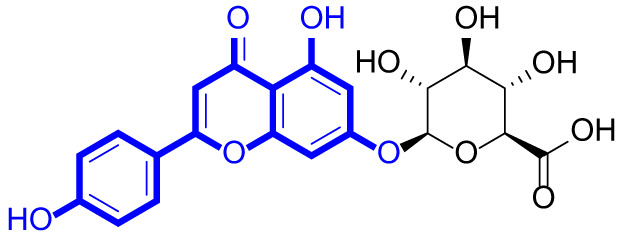	> 40
4	apigenin 7-O-methylglucuronide	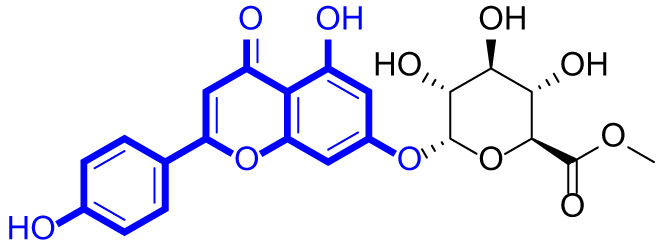	> 40
5	trimethylapigenin	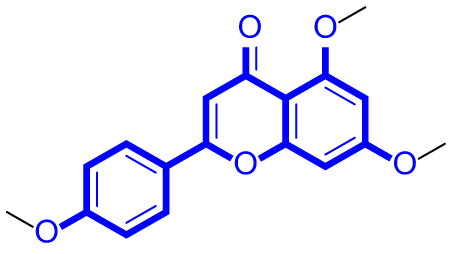	27.63 ± 1.37
6	8,3’-diprenylapigenin	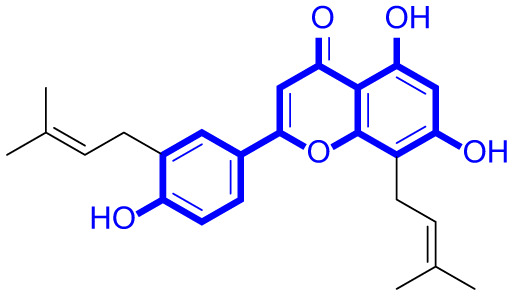	3.60 ± 0.53
7	7,4’-di-O-methylapigenin	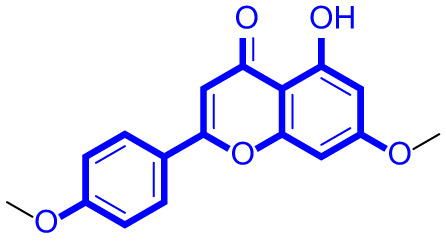	15.93 ± 1.36
8	apigenin 7-diglucuronide	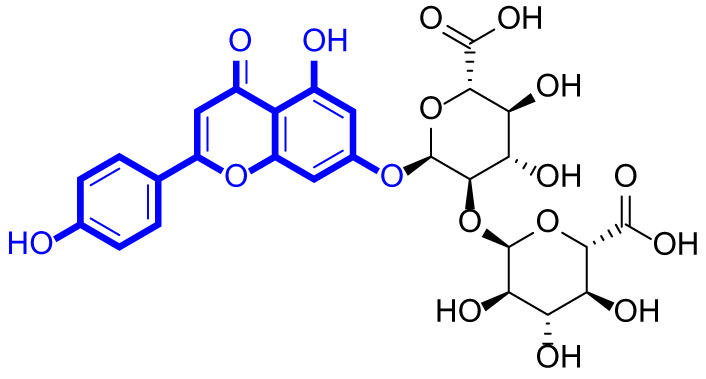	30.51 ± 1.83
9	apigenin triacetate	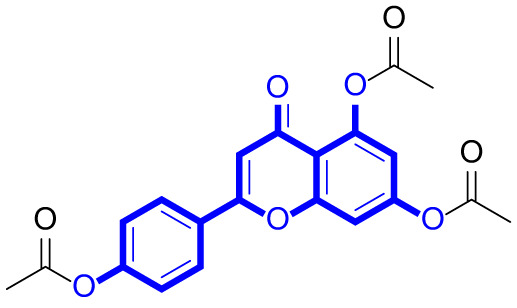	38.67 ± 2.96
10	3’,3’’’-Biapigenin	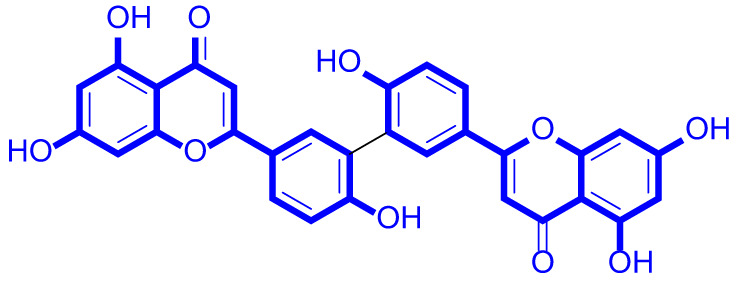	19.27 ± 1.58
11	8-hydroxyapigenin	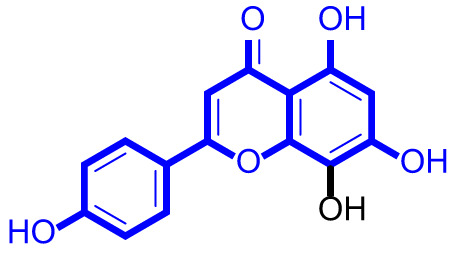	10.09 ± 0.37
12	apigenin 7,4’-diglucoside	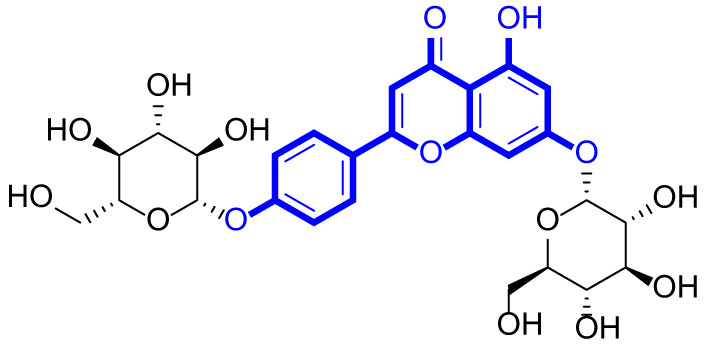	> 40
13	6,8-dimethylapigenin	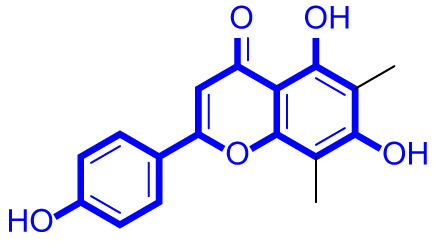	19.76 ± 1.72
14	apigenin 7-O-glucoside	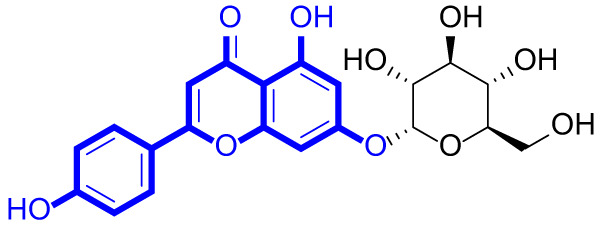	21.39 ± 1.08
15	apigenin 7-O-rutinoside	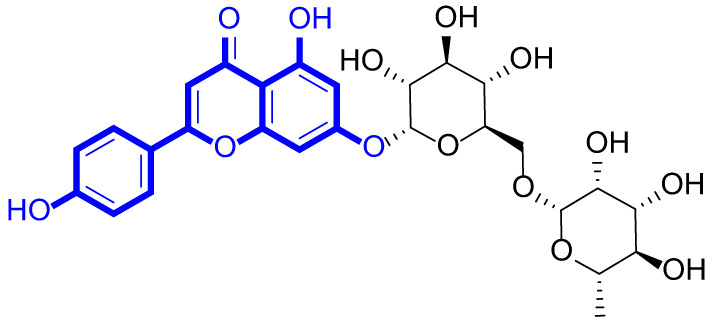	32.67 ± 1.91
16	apigenin 7-neohesperidoside	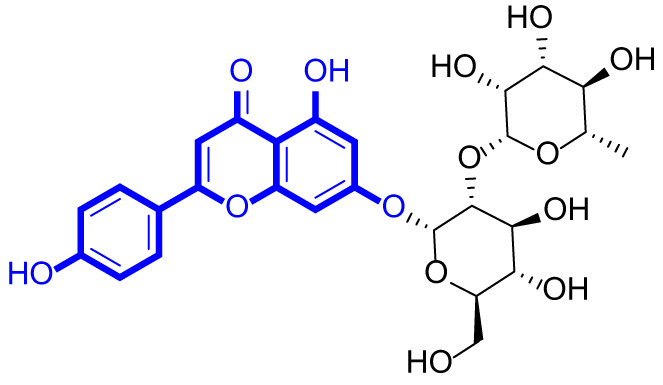	> 40
17	LSD1-IN-24	–	0.37 ± 0.05

### Structure activity relationships of natural apigenin analogues against LSD1

**Fig. 2:**
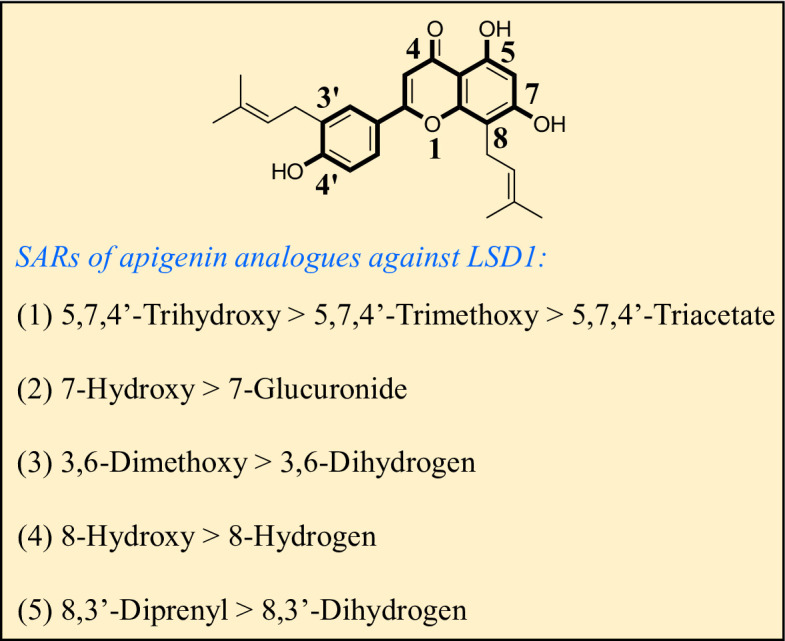
Structure activity relationships of natural apigenin analogues against LSD1.

As shown in Fig. 2, structure activity relationships of natural apigenin analogues against LSD1 were summarized. The replacement of the 5,7,4’-trihydroxy group by the 5,7,4’-trimethoxy group or 5,7,4’-triacetate group resulted in an obvious decrease of activity. Specifically, apigenin showed the inhibitory activity against LSD1 with an IC_50_ value of 13.42 µM (more potent than trimethylapigenin and apigenin triacetate, respectively). Replacement of the 7-hydroxy group (apigenin) with a 7-glucuronide group (apigenin-7-glucuronide) resulted in a decrease for the inhibitory activity. In addition, apigenin 7-O-methylglucuronide, apigenin 7-diglucuronide, apigenin 7,4’-diglucoside, apigenin 7-O-rutinoside and apigenin 7-neohesperidoside all displayed very weak inhibitory effects against LSD1. Changing the 3,6-dimethoxy group (3,6-dimethoxyapigenin) to a 3,6-dihydrogen group (apigenin) led to a decrease for the inhibitory activity against LSD1. With the introduction of an 8-hydroxy group, the inhibitory activity against LSD1 was increased (8-hydroxyapigenin ***VS***. apigenin). In addition, 6,8-dimethylapigenin was more potent than apigenin, indicating that the substituent group on 6,8-position played a significant role. Among all these natural apigenin analogues, 8,3’-diprenylapigenin displayed the most potent inhibitory activity against LSD1, indicating that 8,3’-diprenyl group is better than 8,3’-dihydrogen group.

### 8,3’-diprenylapigenin reversibly and selectively inhibited LSD1 in a concentration-dependent and time-dependent manner

**Fig. 3:**
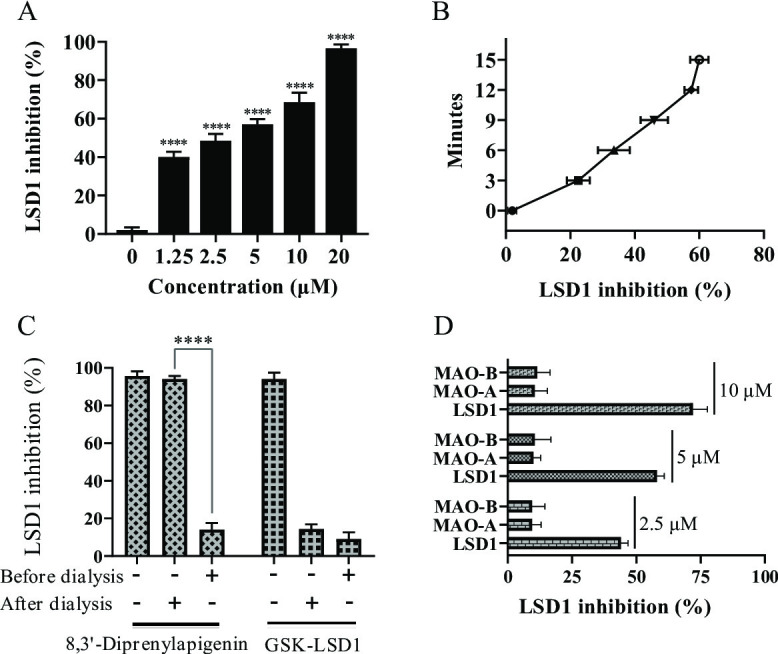
Molecular mechanisms of 8,3’-diprenylapigenin against LSD1. (**A**) 8,3’-diprenylapigenin inhibited LSD1 in a concentration-dependent manner. (**B**) 8,3’-diprenylapigenin inhibited LSD1 in a time-dependent manner. (**C**) The dialysis assay was performed to evaluate its reversibility. (**D**) Inhibitory rates of 8,3’-diprenylapigenin against LSD1 and its homologous proteins MAO-A/B. ^****^P < 0.0001.

Because 8,3’-diprenylapigenin inhibited LSD1 potently, we investigated the molecular mechanisms of 8,3’-diprenylapigenin against LSD1. However, it is not clear whether 8,3’-diprenylapigenin could exert the inhibitory activity against LSD1 in a time-dependent or concentration-dependent manner. From the results in Fig. 3A and B, the inhibitory rates of 8,3’-diprenylapigenin gradually upregulated with the increase of time and concentration, demonstrating that 8,3’-diprenylapigenin inhibited LSD1 in a concentration-dependent and time-dependent manner. In the dialysis assay, GSK-LSD1 as an irreversible LSD1 inhibitor was selected as the control drug. As shown in Fig. 3C, the inhibitory activity of 8,3’-diprenylapigenin against LSD1 was reversed after the dialysis. However, the enzymatic activity of GSK-LSD1 did not show any significant changes during the dialysis. These findings revealed that 8,3’-diprenylapigenin inhibited LSD1 in a reversible manner and it was a new reversible LSD1 inhibitor. LSD1 and its homologous proteins MAO-A/B were important members in the monoamine oxidases family^35–37^. Some LSD1 inhibitors may simultaneously inhibit the enzymatic activity of MAO-A/B, which will lead to toxic effects. Thus, inhibitory effects of 8,3’-diprenylapigenin against MAO-A and MAO-B were also evaluated. As shown in Fig.3D, 8,3’-diprenylapigenin suppressed LSD1 with an inhibitory rate of 72% at 10 µM, while its inhibitory rates against MAO-A and MAO-B were both lower than 12%, indicating that 8,3’-diprenylapigenin was a selective LSD1 inhibitor.

### Molecular docking studies of 8,3’-diprenylapigenin

**Fig. 4:**
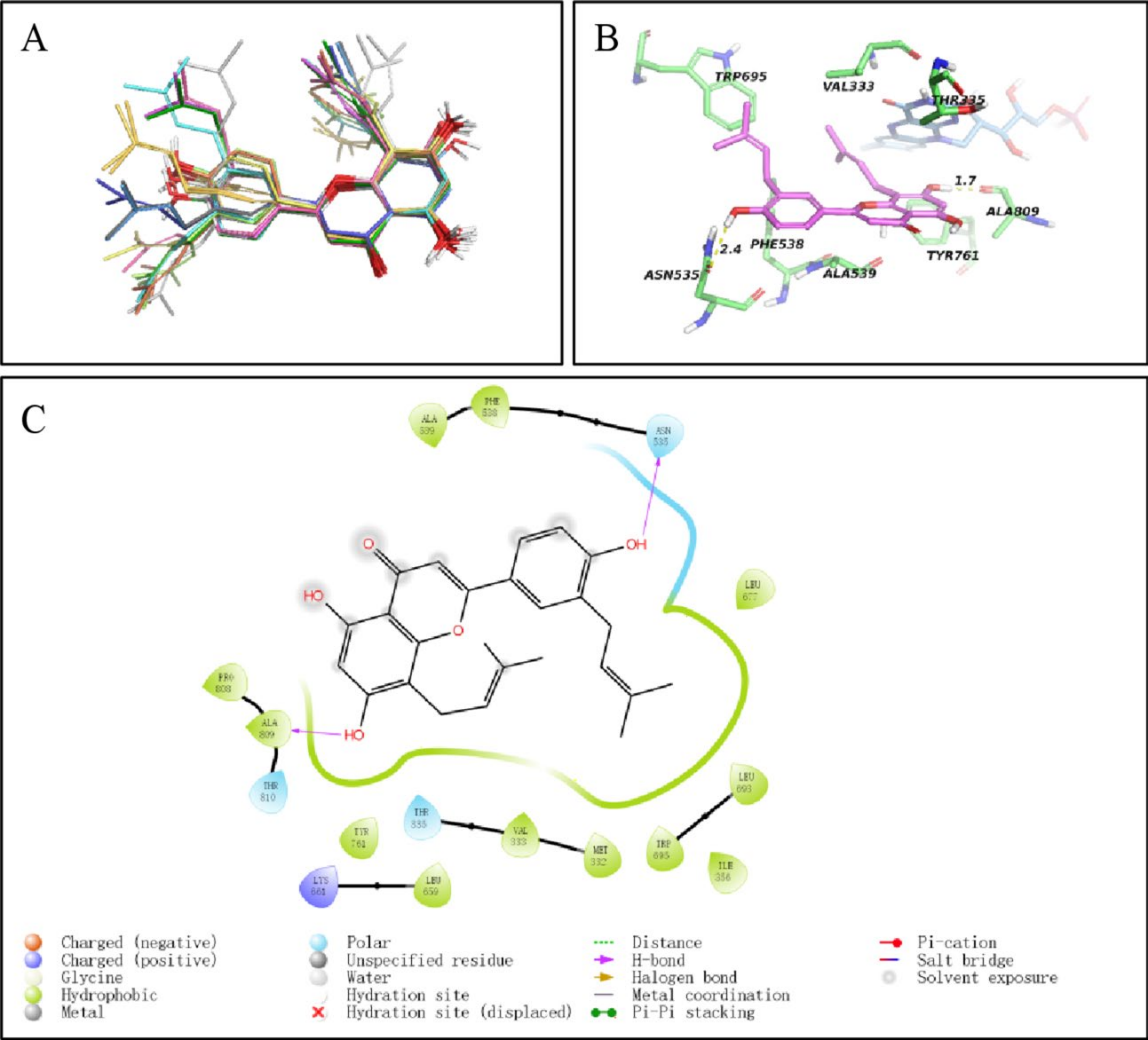
Binding modes of LSD1 and 8,3’-diprenylapigenin. (**A**) 20 Lowest energy binding structures of 8,3’-diprenylapigenin. (**B**) 8,3’-diprenylapigenin formed hydrogen bonds with residues of LSD1. (**C**) Different binding effects of LSD1 and 8,3’-diprenylapigenin.

The binding modes of LSD1 and 8,3’-diprenylapigenin were explored in this work, and the protein structure of LSD1 was used to perform molecular docking studies (PDB code: 2DW4). From the docking results in Fig. 4A and 20 lowest energy binding structures of 8,3’-diprenylapigenin were similar. The docking score of 8,3’-diprenylapigenin toward LSD1 was − 8.71 kcal/mol. As shown in Fig. 4B, 8,3’-diprenylapigenin formed two hydrogen bonds with Asn535 and Ala809 of LSD1. In addition, 8,3’-diprenylapigenin formed the hydrophobic effects with Ala539, Phe538, Leu677, Pro808, Tyr761, Leu659, Val333, Met332, Trp695, Leu693 and Ile356 of LSD1 (Fig. 4C).

### Molecular dynamics simulation of 8,3’-diprenylapigenin

**Fig. 5:**
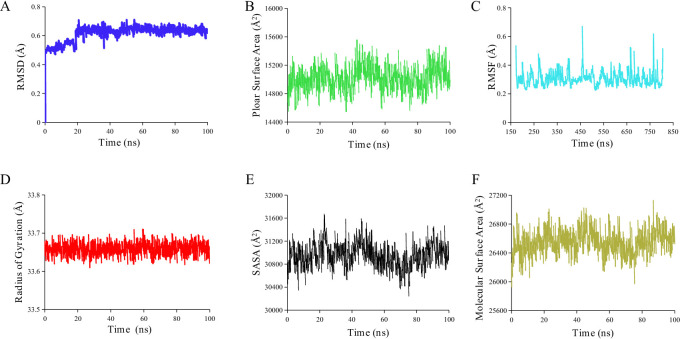
Molecular dynamics simulation of 8,3’-diprenylapigenin. (**A**) RMSD. (**B**) Polar surface area. (**C**) RMSF. (**D**) Radius of gyration. (**E**) Solvent accessible surface area. (**F**) Molecular surface area.

In this work, molecular dynamics simulation of 8,3’-diprenylapigenin were performed. From the results in Fig. 5, the value of RMSD (root mean square deviation) increased rapidly in the first 20 ns and then gradually stabilized, indicating that the protein structure gradually reached a balanced state during the simulation process. The analysis of RMSF (root mean square fluctuation) showed that the fluctuations of some residues in LSD1 were relatively large, while other residues were relatively stable. These results indicated that these residues had the high flexibility during simulation process. Radius of gyration showed that the radius of LSD1 changed over time, reflecting the its compactness. The analytical results of PSA (polar surface area), SASA (solvent accessible surface area) and MSA (molecular surface area) further supplemented the surface characteristic information of LSD1. In addition, the values of van der waals energy, electrostatic energy and non-polar solvation energy were − 62.09 kJ/mol, −19.50 kJ/mol and − 21.03 kJ/mol, respectively.

### Antiproliferative effects of 8,3’-diprenylapigenin against tumoral testicular germ cells, TM3 cells and TM4 cells

**Fig. 6:**
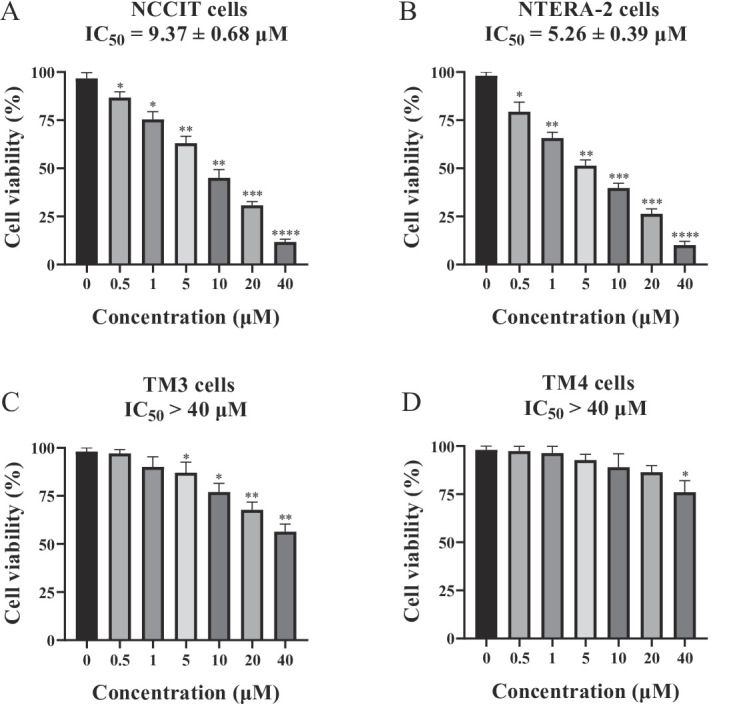
Tumoral testicular germ cell lines and normal testicular cell lines were treated with 8,3’-diprenylapigenin for 48 h. (**A**) Antiproliferative activity against NCCIT cells. (**B**) Antiproliferative activity against NTERA-2 cells. (**C**) Cytotoxic activity against TM3 cells. (**D**) Cytotoxic activity against TM3 cells. ^*^P < 0.05, ^**^P < 0.01, ^***^P < 0.001 and ^****^P < 0.0001.

Antiproliferative activity of 8,3’-diprenylapigenin against tumoral testicular germ cells and its cytotoxic activity against normal testicular cells were evaluated. From the results in Fig. 6, 8,3’-diprenylapigenin inhibited proliferation against tumoral testicular germ cell lines NCCIT and NTERA-2 with IC_50_ values of 9.37 µM and 5.26 µM, respectively. However, 8,3’-diprenylapigenin exhibited very weak cytotoxic activity against normal testicular cell lines (TM3, IC_50_ > 40 µM; TM4, IC_50_ > 40 µM). These results demonstrated that 8,3’-diprenylapigenin had a good selectivity between tumoral testicular germ cells and normal testicular cells.

### 8,3’-diprenylapigenin induced the generation of ROS, inhibited the activity of catalase and decreased the level of ATP in NTERA-2 cells

**Fig. 7:**
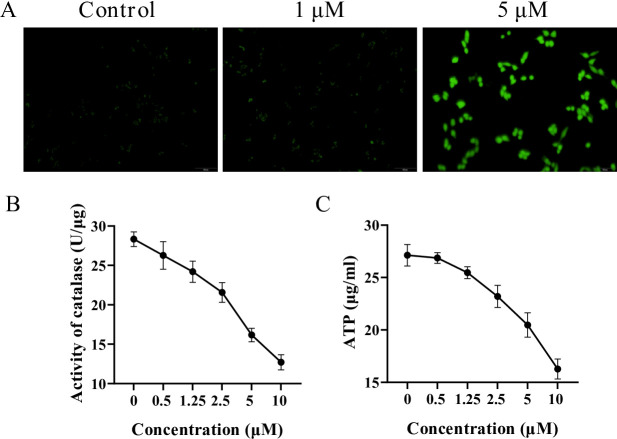
Potential mechanisms of 8,3’-diprenylapigenin on ROS in NTERA-2 cells. (**A**) NTERA-2 cells were treated with 8,3’-diprenylapigenin for 48 h to detect the ROS level. (**B**) Effects of 8,3’-diprenylapigenin on the catalase activity. (**C**) Effects of 8,3’-diprenylapigenin on the ATP level.

**Fig. 8:**
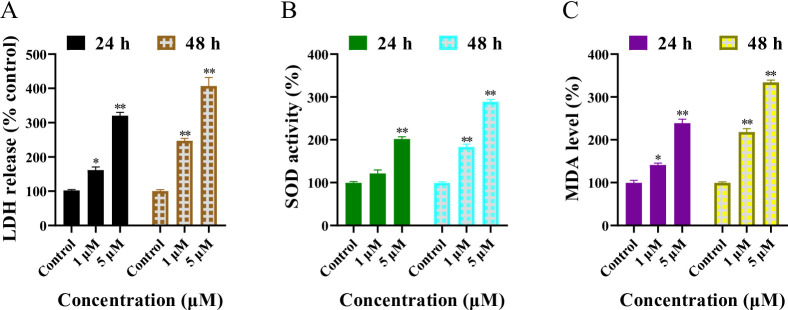
Regulation of oxidative stress-related indicators in NTERA-2 cells treated with 8,3’-diprenylapigenin. (**A**) Effects of 8,3’-diprenylapigenin on the LDH release. (**B**) Effects of 8,3’-diprenylapigenin on the SOD activity. (**C**) Effects of 8,3’-diprenylapigenin on the MDA level. ^*^P < 0.05, ^**^P < 0.01.

Because 8,3’-diprenylapigenin was a novel LSD1 inhibitor with potent antiproliferative effects against testicular germ cell tumors, its potential mechanisms at the cellular level were explored. Recent studies reported that LSD1 inhibitors could generate reative oxygen species (ROS) in cancer cells^38^. From the results in Fig. 7A, 8,3’-diprenylapigenin at 5 µM obviously induced the generation of ROS in NTERA-2 cells. Catalase could degrade hydrogen peroxide and affect the production of ROS in tumor cells^39–41^. As shown in Fig. 7B, 8,3’-diprenylapigenin downregulated the activity of catalase in a concentration dependent manner. Furthermore, 8,3’-diprenylapigenin also concentration-dependently decreased the ATP level in NTERA-2 cells (Fig. 8C).

### 8,3’-diprenylapigenin activated LDH release, increased SOD activity and enhanced MDA levels in NTERA-2 cells

Because 8,3’-diprenylapigenin potently induced the production of ROS, oxidative stress-related indicators such as lactate dehydrogenase (LDH), superoxide dismutase (SOD) and malondialdehyde (MDA) were detected in NTERA-2 cells. When NTERA-2 cells were treated with 8,3’-diprenylapigenin for 24–48 h, LDH release, activities of SOD and contents of MDA were increased with the rise of concentrations (Fig. 8). These results demonstrated that 8,3’-diprenylapigenin promoted the LDH release, upregulated the SOD activity and enhanced the MDA level in NTERA-2 cells in a concentration dependent manner.

## Discussion

Testicular germ cell tumors seriously affect the life and health of men^42^. There is an urgent need for the development of effective drugs to treat testicular germ cell tumors. LSD1 inhibitors exhibit potential inhibitory effects against tumor cells, and LSD1 has become a new target for cancer treatment^43^. In this work, a series of natural apigenin analogues were identified as potential LSD1 inhibitors. Among these apigenin analogues, 8,3’-diprenylapigenin exhibited the best inhibitory activity against LSD1 with an IC_50_ value of 3.60 µM. In addition, 3,6-dimethoxyapigenin also potently inhibited LSD1 with an IC_50_ value of 8.75 µM. Replacement of the 7-hydroxy group from apigenin with a 7-glucuronide group resulted in a decrease for the inhibitory activity, indicating that 7-hydroxy group displayed a significant role for the inhibitory activity. Furthermore, we summarized the structure activity relationships of natural apigenin analogues against LSD1. These findings will provide the structural guidance for the design of novel and effective LSD1 inhibitors.

The molecular mechanisms of 8,3’-diprenylapigenin against LSD1 were investigated. It inhibited LSD1 in a concentration-dependent and time-dependent manner. Irreversible LSD1 inhibitors have shown several immune system side effects and blood system side effects in clinical applications^44^. Therefore, in order to provide more options for patients with cancer, researchers are currently actively seeking new reversible inhibitors. From results in the dialysis assay, we found that 8,3’-diprenylapigenin inhibited LSD1 in a reversible manner and it was a new reversible LSD1 inhibitor. Importantly, 8,3’-diprenylapigenin displayed negligible inhibition of MAO-A/B, indicating that 8,3’-diprenylapigenin was a selective LSD1 inhibitor. Based on molecular docking studies, 8,3’-diprenylapigenin formed two hydrogen bonds with Asn535 and Ala809 of LSD1. Molecular dynamics simulation of 8,3’-diprenylapigenin were performed, and the values of van der waals energy, electrostatic energy and non-polar solvation energy were − 62.09 kJ/mol, −19.50 kJ/mol and − 21.03 kJ/mol, respectively.

Although LSD1 is overexpressed in NCCIT and NTERA-2 cell lines, few studies have reported LSD1 inhibitors with anti-testicular germ cell tumor activity^45^. In this work, 8,3’-diprenylapigenin exhibited potent antiproliferative activity against tumoral testicular germ cell lines NCCIT and NTERA-2 with IC_50_ values of 9.37 µM and 5.26 µM, respectively. Interestingly, 8,3’-diprenylapigenin exhibited very weak cytotoxic activity against normal testicular cell lines TM3 and TM4. These findings illustrated that 8,3’-diprenylapigenin had a good selectivity between tumoral testicular germ cells and normal testicular cells. Therefore, 8,3’-diprenylapigenin has the potential to be developed into a clinical candidate drug.

To the best of our knowledge, there are currently no relevant literature reports on natural apigenin analogues as LSD1 inhibitors against testicular germ cell tumors. The potential mechanisms of 8,3’-diprenylapigenin at the cellular level were investigated. The pharmacological inhibition of LSD1 leads to the accumulation of superoxide, thereby exerting a promoting effect on oxidation^46^. In NTERA-2 cells, 8,3’-diprenylapigenin could induce the generation of ROS, inhibit the activity of catalase and decrease the level of ATP. Moreover, 8,3’-diprenylapigenin increased the release of LDH, upregulated the activity of SOD and enhanced the level of MDA. Therefore, the inhibition of LSD1 disrupts the redox balance, leading to the eventual death of tumoral testicular germ cells.

## Conclusions

In this work, a series of natural apigenin analogues were evaluated for their inhibitory activity against LSD1. Among them, 8,3’-diprenylapigenin displayed the most potent inhibitory activity against LSD1 with an IC_50_ value of 3.60 µM. Structure activity relationships indicated that 7-hydroxy is a dominant group, and 7-hydroxy of 8,3’-diprenylapigenin formed a hydrogen bond with Ala809 of LSD1. At the enzymatic level, 8,3’-diprenylapigenin reversibly and selectively inhibited LSD1 in a concentration-dependent and time-dependent manner. It inhibited proliferation against tumoral testicular germ cell lines NCCIT and NTERA-2 with IC_50_ values of 9.37 µM and 5.26 µM, respectively. At the cellular level, 8,3’-diprenylapigenin induced the generation of ROS, inhibited the activity of catalase and decreased the level of ATP in NTERA-2 cells. In addition, it also activated LDH release, increased SOD activity and enhanced MDA levels in NTERA-2 cells. So far, there have been no literature reports on apigenin analogues as selective LSD1 inhibitors with antiproliferative activity against tumoral testicular germ cells. This work could provide the promising bioactive skeleton and leading compounds for designing more potent anti-TGCT agents.

## Supplementary Information

Below is the link to the electronic supplementary material.

Supplementary Material 1

## Data Availability

The datasets used and/or analysed during the current study available from the corresponding author on reasonable request.
