# Supplementary material for: Discovery of natural apigenin analogues as lysine-specific demethylase 1 inhibitors against tumoral testicular germ cells
Source: Sci Rep. 2026 Mar 2;16:8917. doi: 10.1038/s41598-026-42263-y (PMC12988231; doi:10.1038/s41598-026-42263-y)
Supplement: Supplementary file 1 — Supplementary Material 1 [file 41598_2026_42263_MOESM1_ESM.docx]

**Discovery of natural apigenin analogues as lysine-specific demethylase 1 inhibitors against tumoral testicular germ cells**

Li-Wei Sun^1*^, Meng Zhang^1^, Cai-Fang Li^1^, Cong Wang^1^, Yang Li^1^

^1^Xinxiang Central Hospital, The Fourth Clinical College of Henan Medical University, Xinxiang 453000, China

^*^Corresponding author: [xxszxyyslw@outlook.com](mailto:Xxszzyyslw@outlook.com) (Li-Wei Sun)

 **Fig. S1**. The discovery strategy of natural apigenin analogues as LSD1 inhibitors
